# Prevalence of pulmonary tuberculosis before and after soil dust in Khuzestan, southwest Iran

**Published:** 2014

**Authors:** Seyed Mohammad Alavi, Pejman Bakhtiyariniya, Mehdi Eghtesad, Shokrollah Salmanzadeh

**Affiliations:** 1 Health Research Institute, Infectious and Tropical Diseases Research Center, Joundishapur University of Medical Sciences, Ahvaz, Iran; 2 Khuzestan Health Center, Ahvaz Joundishapur University of Medical Sciences, Ahvaz, Iran

**Keywords:** Pulmonary tuberculosis, Soil exposure, National Tuberculosis Program, MDR-TB, Iran

## Abstract

***Background: ***Soil dust has been debated about its effects on public health and the challenge is brought about tuberculosis (TB). The purpose of this study was to investigate the influence of soil dust on pulmonary tuberculosis (PTB) prevalence and its control indices.

***Methods:*** The medical files of patients in Khuzestan Health Center were reviewed. The control group included the PTB patients registered from 2005 to 2006 (before soil dust), and case group consisted of PTB patients who were registered from 2007 to 2010 (after soil dust exposure). The diagnosis of tuberculosis was based on National Tuberculosis Program (NTP).

***Results:*** The mean age of control and case group was 42 (18-80) years and 40 (13-99) years, respectively. The prevalence of pulmonary TB in the control and case group was 537 (12.5 per 100000 population) and 465 (11.0 per 100000 population), respectively. Exposure to dust did not increase the prevalence of TB. The prevalence was higher in women than men (298, 41.8% vs. 336, 48.2%), in children than adult group (31, 4.3% vs. 53, 7.3%), in urban than rural inhabitants (448, 63% vs.496, 71.1%) and in family contacts than solitary contamination (60, 8.4% vs. 97, 13.9%). The rate of treatment failure, TB relapse, and MDR-TB in controls and cases were (1.4%, 1.4%, 1%) and (7%, 5.5%, 4.6%), respectively. Dust exposure had significant effect on treatment outcome.

***Conclusion: ***Although soil dust exposure had no significant effect on TB prevalence, but significantly affected the prevalence of TB respecting to age, sex, residential area and closed contact. In addition resulted in more treatment failure, development of MDR TB and relapse.

Dust phenomenon in the recent years has been debated about the effects of this phenomenon on health and the challenge is brought. According to the Iranian environment organization, the level of air pollution due to the soil dust in 2011 was more than 2900 µg/m^3^ which was 19 times the standard limit (1). When the concentration of these particles (soil dust particle) is less than 150 µg/m^3^, air is defined as healthy; 150-420 µg/m^3^, unhealthy and more than 420 µg/m^3^ considered as hazardous (2). According to the Khuzestan Meteorological Agency, since 2007 in the city of Ahvaz, the center of Khuzestan province, over 323 days of crisis in air pollution is caused by these particles (3). The increase of each 10 µg/m^3^ of the particles exceeded 1% increase in mortality in the population. It is estimated about 1.34 million death occurred due to the air pollution in this period of time (4). Drought, drying wetlands and rivers resulting desertification in the west of country from western neighbors especially Iraq have had a lot of effects on the soil dust phenomenon (5). 

The soil dust contains biological particles, organic compounds and metals and is impressive on the incidence of respiratory diseases (6, 7). Breathing air containing dust over time can cause pathological changes in the lungs. The effect of silica, cotton, wool, weaving mills, coal mine is a known issue on the incidence of respiratory diseases mentioned in the most medical reference books (8-10). The association of pulmonary tuberculosis and air pollution sources such as silica containing dust, coal mine dust, carpet and cotton dust, and smoking is a known subject and a lot of articles have been written on this topic (8, 9, 11-15). Since the occurrence of this event, the question has preoccupied the regional health officials’ minds; whether the dust has effect on TB control and the trend of the disease (10). Considering the endemicity of tuberculosis (16, 17), the specific geopolitical condition, holy shrine pilgrims traveling route (pilgrimage infallible Imams in Iraq) and input marine transit, Khuzestan has a significant status for tuberculosis control in the country. To the best of our knowledge, a similar study has not been performed in this field in the region and even elsewhere. The aim of this study was to investigate the effect of dust on tuberculosis and its control indices. The result of this study will help the health policymakers in the region for better TB control.

## Methods

**Study design and data: **In a retrospective study based on the existing data in Khuzestan Health Center (KHC), from 2005 to 2010, tuberculosis patients were studied. 

**Place of study and population: **Tuberculosis cases over the 6-year period in Khuzestan (the southwestern province of Iran) with a population of about 4.5 million people living in 23 cities (except Dezful) were reviewed.

**Data collection: **The data extracted from the patients' records include the demographic characteristics, clinical symptoms, laboratory and radiological findings and its control measures such as: response to treatment, treatment failure, relapse and mortality in both groups were compared. Average values in both groups were compared.

**Sample size: **The medical files of patients in Khuzestan Health Center were reviewed. Given that 2007 is the beginning of this phenomenon, the patients were categorized into two groups. The control group, included all the patients registered from 2005 to 2006 (before soil dust occurrence). The case group, included all the patients registered from 2007 to 2010 (after soil dust occurrence). Inclusion criteria were documented TB, diagnosed based on National Tuberculosis Program (NTP) (18).

**Definitions: **Smear positive pulmonary tuberculosis (SPPT): Cases with at least two smears of sputum positive for acid fast bacillus (SSP-AFB) or, a chest radiography suggestive of tuberculosis plus one SSP-AFB or, sputum culture positive for M.tuberculosis and one SSP-AFB were defined as smear positive pulmonary tuberculosis (PTB+).

**Smear negative pulmonary tuberculosis (SNPT):** Cases with clinical finding suggestive of TB plus three sputum smears negative for AFB (SSN-AFB) after two weeks of antibiotic therapy plus C-X-ray (suggestive TB) were defined as smear negative pulmonary tuberculosis (PTB-). Other diagnostic criteria were cerebro spinal fluid analysis for TB meningitis and CT-scan and microbial study for milliary or extrapulmonary tuberculosis.

**Cured:** After 2-5 months after the start of treatment, the patient’s sputum has been converted from AFB-positive to AFB-negative.

**Treatment failure:** After 5 months or more after the start of treatment, the patient’s sputum still remains positive, or within the same time changes from negative to positive again. Or the treatment of smear-negative cases in the beginning, but after two months of treatment, sputum examination became positive. 

**Relapsed TB:** A patient now with sputum smear positive for AFB admitted in the past, and received anti-tuberculosis treatment from a physician of any forms of tuberculosis, and was categorized as completed or cured previously. 

**Multidrug resistant TB (MDR-TB):** Cases with positive drug resistant tests against both rifampicin and isoniazid were investigated in referral TB laboratory centers in Shiraz or Tehran. A patient under treatment is said to be transferred out, if he/she is transferred to another unit, to continue the treatment is said to be transferred in, if he/she is transferred to our unit. HIV infection was diagnosed on the basis of enzyme-linked immunosorbent assay (ELISA) and western blotting (8). 

**Statistical analysis: **SPSS software system, Version 16 was used to derive the descriptive statistics and in subsequent multivariable analyses, chi square and Fisher’s exact tests were used to compare the data in both groups. Chi square test was used to compare the qualitative variables in this study. The differences with p-values less than 0.05 were considered significant.

## Results

Of the total 5764 people who referred each year to health care setting with chronic cough (more than 2 weeks) in the period before soil dust phenomena, 537 (12.5 per 100000 population) have been diagnosed with pulmonary tuberculosis. It means that every 10 people suffering from coughing more than 2 weeks approximately one case has pulmonary tuberculosis. Of the total 7625 people annually, with coughing more than 2 weeks in a period after soil dust, 465 (11.0 per 100000 population) have been diagnosed as pulmonary tuberculosis, meaning that in every 16 people suffering from coughing more than 2 weeks, approximately one case has pulmonary tuberculosis. In the period before the dust, an annual average of 712 people had tuberculosis, among them, 175 had extra pulmonary tuberculosis, 415 SPPT and 122 SNPT, respectively. In the period after the soil dust, an average of 698 people had tuberculosis including 233 patients with extra pulmonary tuberculosis, 344 SPPT and 121 SNPT, respectively. The mean age of pulmonary TB patients before and after dust was 42 (18-80) years and 40 (13-99) years, respectively. TB infection indicators and demographic indices in the studied patients are shown in tables 1 and 2.

**Table 1:** Demographic characteristics of tuberculosis patients before and after occurrence of soil dust in Khuzestan

**P-value**	**After** **N (%)**	**Before** **N (%)**	** Soil dust** **Variables **
0.01 0.87 0.59 0.32	53 (7.3) 336 (48.1) 193 (27.6) 116 (17)	31 (4.3) 346 (48.6) 206 (28.9) 133 (18.2)	**Age (years)** <15* 15-40 41-60 >60
0.01	362 (51.8) 336 (48.2)	414 (58.2) 298 (41.8)	**Sex ** Male* Female
0.001	496 (71.1) 202 (28.9)	448 (63) 264 (37)	**Residential area** Urban* Rural
<0.0001	42 (6)	91 (12.8)	Imprisonment
0.61	16 (2.3)	20 (2.8)	HIV infection
0.001	97 (13.9)	60 (8.4)	TB contact history*
0.25	681 (97.6) 17 (2.4)	701 (98.4) 11 (1.6)	**Nationality** Iranian Non-Iranian
0.02	596 (85.3)	584 (82)	Low socioeconomic*
	698 (100)	712 (100)	Total TB cases

* statistically significant

**Table 2:** Infection indicators of tuberculosis, before and after the occurrence of soil dust in Khuzestan

** Soil dust** **Variables **	**Before** **N (%)**	**After** **N (%)**	**Pvalue**
Positive sputum PTB	415 (77.3)	344 (73.9)	0.23
Negative sputum PTB	122 (22.7)	121 (26.1)	
Conversion (Pos.to Neg.)*	312 (75.1)	227 (65.9)	0.003
Conversion (Neg. to Pos.)	6 (4.9)	7 (5.8)	0.78
Total PTB	537 (100)	465 (100)	

* statistically significant

Due to increase in patients with chronic cough in post soil dust era, sputum testing also increased. On average, for each patient with prolonged cough, sputum test was done 3 times, whereas, before dust era this average was 2 times respectively. As table 2 shows, the dust has no effect on the number of patients. Despite the stagnant population (4278671 and 4231392 medium population of Khuzestan before and after the soil dust), the total number of TB patients was not significantly different. Although soil dust has no effect on TB prevalence, but had on age, sex, urbanization and exposure outcome. More women than men, more children than any other age group, more urban habitants than rural settlers and more family contacts had been sensitized to TB and became ill. Dust also has negative effects on sputum clearance of mycobacterium at the end of the second month of treatment (table 2). Air pollution caused by soil dust has increased the rate of treatment failure, TB relapse, and the probability of occurrence of MDR-TB (table 3). 

**Table 3:** Treatment/control indicators of tuberculosis, before and after occurrence of soil dust in Khuzestan

** Soil dust** **Variables**	**Before** **N (%)**	**After** **N (%)**	**P-value**
Cured	307 (73.9)	256 (74.4)	0.93
Completed treatment	26 (6.2)	22 (6.4)	1.0
Failed treatment*	6 (1.4)	24 (7)	0.0001
Relapsed TB*	6 (1.4)	19 (5.5)	0.001
Death of PTB	29 (7)	22 (6.4)	0.77
Transferred out	12 (2.9)	15 (4.3)	0.32
Transferred in	8 (1.9)	6 (1.7)	1.0
MDR-TB*	4 (1)	16 (4.6)	0.002
Total PTB	415 (100)	344 (100)	

* statistically significant

**Figure 1:**
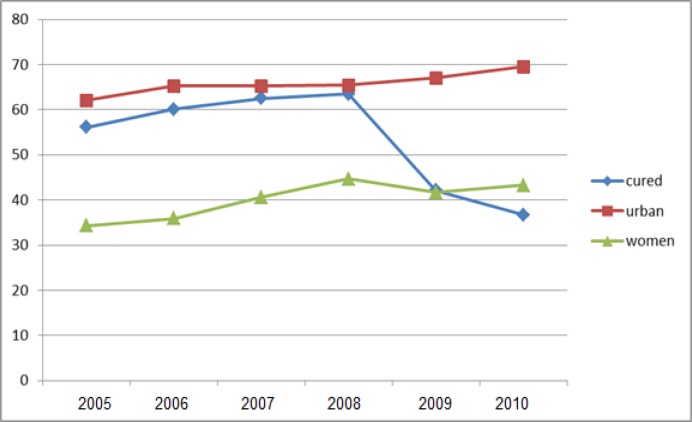
Trend of cured, urban and female tuberculosis patients in Khuzestan of 6 years study

**Figure 2:**
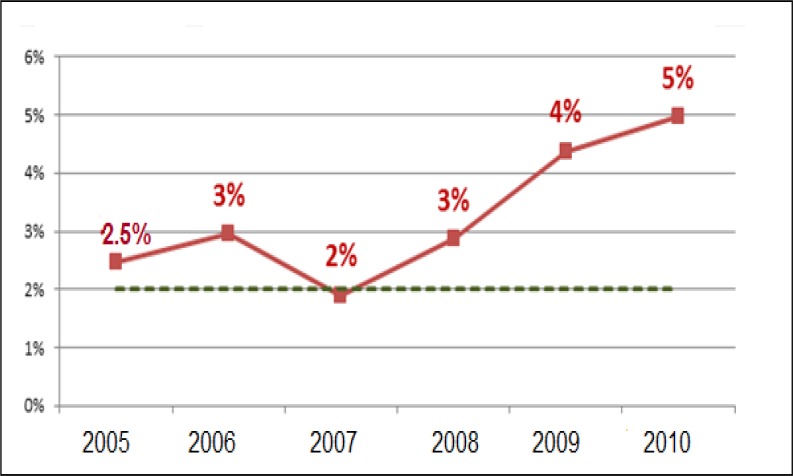
Tuberculosis treatment failure trend in Khuzestan of 6 years study

The trend of treatment failure, cured, urban and female tuberculosis patients in Khuzestan in the 6 years study is shown in figures 1 and 2. As shown in these figures, there are upward trends for MDR-TB and pulmonary TB in children and women in the community and a downward trend for cure rate.

## Discussion

The present study revealed that the number of patients with cough duration of more than 2 weeks had increased due to air pollution caused by soil dust since 2007 in Khuzestan province. Inhalation of the fine soil particles results in respiratory insufficiency and the patients are prone to respiratory diseases such as bronchitis or bronchial asthma (9). Increased time of exposure to silica in inhaled soil dust is associated with the increased risk of pulmonary fibrosis and presentation of respiratory sign and symptoms such as chronic cough and dyspnea (9, 14, 19). In spite of the increased number of patients with chronic cough due to soil dust phenomena, the number of patients with pulmonary TB has not increased. Indeed, soil dust did not have an effect on the prevalence of tuberculosis. This finding is contrast to the results of previous studies (8-11, 13-15, 20). Singh et al. reported that the inhalation of air polluted by coalmine dust with concentration of 155-393 µg/m^3^ has increased the number of patients with pulmonary tuberculosis (15). Mahapatra et al. had explained that exposure to silica increased the risk of pulmonary tuberculosis (20). Increased period of exposure time was associated with more pulmonary TB patients (14). Other investigators reported similar results (8-10). Duffield et al. reported that the risk of acquiring active tuberculosis had increased by nearly seven-folds due to silica exposure in stone crushing mills in India. They believe that soil dust storm that has recently emerged in the west Asian countries is suspected to be an airborne silica exposure for the inhabitants of these areas (10).

The reasons for these differences in our results with other studies were not clearly determined by us. We believe that some differences in study design, kind of air pollution sources and duration of exposure may interfere to our discussion. Our patients were exposed to fine dust particles with unknown chemical and microbial components, whereas the patients in other studies were exposed to silica or coalmine dust which was a known cause of occupational risk factors for tuberculosis (9, 13-15, 20). The population in this study was revisited from a general population, whereas, in other studies the employees in certain occupations were studied. In our study, the period of exposure to soil dust was 3-4 years, whereas, in other studies it was 10 years or more (14). Our study showed that soil dust had increased the number of female with pulmonary TB. Although female to male ratio was constantly unchanged in general population during this period, but the prevalence of pulmonary TB increased in female patients compared with male patients. We found no similar studies to compare these results, but the studies performed in silicosis patients showed that men more than women are at risk of pulmonary TB. Some issues may be considered for explanation. First; both tissue immune response and cellular immunity are impaired in women more than in men in response to air pollution sources such as silica or smoking, second; men are more involved than women in occupational dust exposure (8, 9, 21).

The present study showed a significant effect of soil dust on TB prevalence among children. The prevalence rate of pulmonary TB for children under 15-years of age changed from 4.3% to 7.3% during this period of time. The effect of dust on TB prevalence among urban habitants was more than in rural cases. Large crowded population in big cities especially in families with more population in contact with active TB cases is associated with the higher risk of tuberculosis. Other factors such as poor nutrition, homelessness and poverty in big cities may contribute in TB infection and is an important bias for the effect of dust on tuberculosis (8).

This study revealed that secondary TB in closed contacts had increased in dusty condition. This finding confirms our discussion on the higher frequency of TB in crowded families with index TB case. We found that soil dust may diminish conversion rate at the end of 2 month anti-TB treatment. Conversion rate is a useful prognostic indicator of success in TB control program. Decreased in conversion rate is associated with the high probability of treatment failure and emerging of microbial resistance to anti-TB drugs. Indeed delay in conversion from positive AFB-sputum to negative AFB-sputum blocks pulmonary *M.tuberculosis* clearance and prolongs the infection period, and more in contact individuals are at the risk of TB infection (8,18). Our study showed a worse effect of soil dust on treatment outcome. Failure rate increased from approximately 2.5% to 5% during this period. 

As a result, the patients remain infectious, and more people will be at risk of exposure to *M.tuberculosis* especially the multidrug resistant organisms. Treatment outcomes have not been discussed in silicosis TB patients in comparison with non-silicosis TB patients in literature or published studies (8, 9, 14). According to the National TB program in Iran, the expected failure rate is less than 2%, so dust phenomenon has made regional TB program far from the standard of the country (18).

The present study declared that both the relapse of tuberculosis and the emerging of MDR-TB are under the influence of soil dust. Increased in number of patients with relapsed pulmonary TB and MDR-TB patients during this period indicates that air pollution caused by soil dust threatens the National Anti-TB program in Khuzestan as well as the other 11 western and southern provinces throughout the country. MDR-TB means M.tuberculosis is resistant to at least 2 major anti TB drugs; rifampicin and isoniazid (8, 18). The increased rate of MDR-TB in our region highlights the state health policymakers to be aware of this important problem involving TB program. More investigations in various fields such as molecular biology, in vivo drug susceptibility tests, anti-TB drug efficacy, and immunology are needed to elucidate the reasons for these results.

*Strengths and limitations of the study: *All cities and villages in the region of the study as well as sex, age group and residency were included. The limitations are that the chemical and biological characteristics and components of fine particles were not clearly defined, short duration of exposure to dust was not enough to detect the pathological effect of dust on studied population, the design of the study (before and after studies) with the probability of bias due to confounding factors (e.g. variation in public socio-economic, health condition, and natural trend in disease). Further studies on soil dust in Khuzestan are recommended.

In conclusion**,** although, soil dust had no obvious effect on TB prevalence, but showed a significant effect on age, sex, residential area, closed contact, failure treatment, relapse and microbial resistance. The prevalence of pulmonary TB increased in children below 15 years, women, urban population, and household contact. Likewise treatment failure, relapsed of TB and MDR-TB also increased.

## Notes

**Financial support:** This study was supported by the Infectious and Tropical Research Center of Ahvaz Joundishapur University of Medical Sciences.

## References

[1] Khaksar E Khuzestan air pollution 20 times normal status. Khuzestan Environmental Organization Report, 2011. http://www.jahannews.com.

[2] Khaksar E Dust soil in Khuzestan 13 times over normal standard. Khuzestan environmental organization report, 2011. http://www.tsdle.ir.

[3] Shafikhani N 323 critical days in Khuzestan. Khuzestan meteorological bureau report, 2011. http://www.farsnews.com/1390/0705000977.

[4] Khosravi N Air pollution increasing illness in anxious.Shahrekord Health Center report, 2011. http://www.farsnews.com/1390/026000417.

[5] Gerivani H, Lashkaripour GR, Ghafoori M, Jalali N (2011). The sources of dust storm in Iran, A case study based on geological information and rain fall data. Carpanthian J Earth Environ Sci.

[6] Kenzaka T, Sueyoshi A, Baba T (2010). Soil microbial community structure in an Asian dust source region (Loess plateau). Microbes Environ.

[7] Griffin DW (2007). Atmospheric movement of microorganisms in clouds of desert dust and implications for human health. Clin Microbiol Rev.

[8] Fitzgerald DW, Sterling TR, Haas DW, Mandell GL, Bennett JE, Dolin R ( 2010). Mycobacterium tuberculosis. Principle and practice of infectious diseases.

[9] Pappas PG, Mandell GL, Bennett JE, Dolin R (2010). Chronic pneumonia. Principle and practice of infectious diseases.

[10] Duffield BJ, Young DA (1985). Survival of Mycobacterium bovis in defined environmental conditions. Vet Microbiol.

[11] Gupta MN (1959). Epidemiology of tuberculosis with special references to dusty industries. Ind J Tub.

[12] Mo J, Wang L, Au W, Su M (2014). Prevalence of coal workers pneumoconiosis in China: A systemic analysis of 2001-2011 studies. Int J Hyg Environ Health.

[13] Leung CC, YuIT, Chen W (2012). Silicosis. Lancet.

[14] Milovanovic A, Nowak D, Hering KC (2011). Silicotuberculosis and silicosis as occupational diseases: Report of two cases. Srp Arh CelokLek.

[15] Singh G, Pal A, Khoiyanbam RS (2009). Impact of mining on human health in and around Jhansi, Bundelkhand region of Uttar Pradesh, India. J Ecophysiol Occupt Health.

[16] Arsang SH, Kazemnejad A, Amani F (2011). Epidemiology of tuberculosis in Iran (2001-08). J Gorgan Univ Med Sci.

[17] Metanat M, Sharifi-Mood B, Alavi naeini R, Aminianfar M (2012). The epidemiology of tuberculosis in recent years: Reviewing the status in south-eastern Iran. Zahedan J Res Med Sci.

[18] Mirhaghani L, Nasehi M (2002). Ministry of Health National tuberculosis program in Iran.

[19] Mohebbi I, Aslan Abadi N, Booshehri B, Zubeyri T, Ghavam F (2007). Rapidly progressive silicosis. Tanaffos.

[20] Mahapatra H, Goswami S, Dey D (2010). Coalmine dust concentration and rate of tuberculosis infection around I valley coal field, Orissa, India. J Environ Biol.

[21] Alavi SM, Ershadian S (2009). Association between cigarette smoking and pulmonary tuberculosis. Pak J Med Sci.

